# Effectively Modulating Oxygen Vacancies in Flower-Like *δ*-MnO_2_ Nanostructures for Large Capacity and High-Rate Zinc-Ion Storage

**DOI:** 10.1007/s40820-023-01194-3

**Published:** 2023-10-07

**Authors:** Yiwei Wang, Yuxiao Zhang, Ge Gao, Yawen Fan, Ruoxin Wang, Jie Feng, Lina Yang, Alan Meng, Jian Zhao, Zhenjiang Li

**Affiliations:** 1 College of Materials Science and Engineering, Qingdao University of Science and Technology, Qingdao, 266042 Shandong People’s Republic of China; 2 College of Chemistry and Molecular Engineering, Qingdao University of Science and Technology, Qingdao, 266042 Shandong People’s Republic of China

**Keywords:** Znic-ion battery, *δ*-MnO_2_ cathode materials, Oxygen vacancy modulation, Large specific capacity, High-rate capability

## Abstract

In recent years, manganese-based oxides as an advanced class of cathode materials for zinc-ion batteries (ZIBs) have attracted a great deal of attentions from numerous researchers. However, their slow reaction kinetics, limited active sites and poor electrical conductivity inevitably give rise to the severe performance degradation. To solve these problems, herein, we introduce abundant oxygen vacancies into the flower-like *δ*-MnO_2_ nanostructure and effectively modulate the vacancy defects to reach the optimal level (*δ*-MnO_2−*x*_−2.0). The smart design intrinsically tunes the electronic structure, guarantees ion chemisorption–desorption equilibrium and increases the electroactive sites, which not only effectively accelerates charge transfer rate during reaction processes, but also endows more redox reactions, as verified by first-principle calculations. These merits can help the fabricated *δ*-MnO_2−*x*_−2.0 cathode to present a large specific capacity of 551.8 mAh g^−1^ at 0.5 A g^−1^, high-rate capability of 262.2 mAh g^−1^ at 10 A g^−1^ and an excellent cycle lifespan (83% of capacity retention after 1500 cycles), which is far superior to those of the other metal compound cathodes. In addition, the charge/discharge mechanism of the *δ*-MnO_2−*x*_−2.0 cathode has also been elaborated through ex situ techniques. This work opens up a new pathway for constructing the next-generation high-performance ZIBs cathode materials.

**Supplementary Information:**

The online version contains supplementary material available at 10.1007/s40820-023-01194-3.

## Highlights

- The flower-like* δ*-MnO_2_ nanostructures with controlled oxygen vacancies as an extraordinary ZIBs cathode are innovatively developed.
- The cathode can present large capacity and high-rate zinc-ion storage.
- DFT analysis substantially unveils the effects of various vacancy concentrations on their electrochemical performances.

**Supplementary Information:**

The online version contains supplementary material available at 10.1007/s40820-023-01194-3.

## Graphical abstract

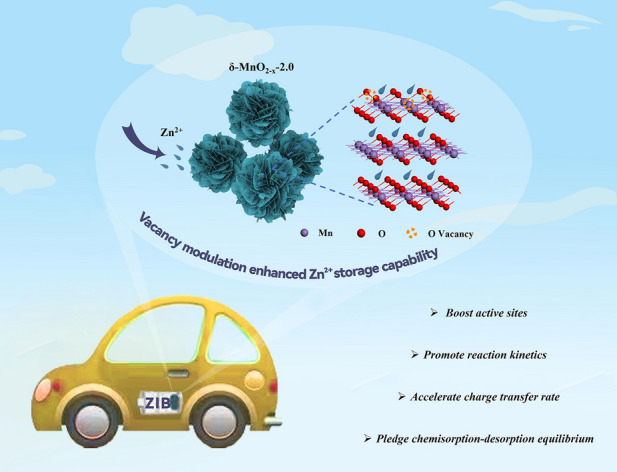

## Introduction

Developing low-cost, high-safety and large energy density energy storage devices is extremely necessary for large-scale renewable energy storage system [1–6], of which aqueous zinc-ion batteries (ZIBs) are expected to be the potential devices due to the superior energy densities, suitable redox voltage, affordability and environmental friendliness [7–9]. The cathode materials are important components that determine the electrochemical performances of ZIBs [10–14]. Among various cathode materials, the transition metal oxides, especially manganese dioxide (MnO_2_), have become highly competitive candidates because of its high theoretical capacity, multiple crystal structures and wide operating potential [15–17]. However, in the previous studies, its actual measured specific capacity was lower than the theoretical value, and the rate capability at the high current density was also poor, which possibly results from the inherent inferior electrical/ionic conductivity and the sluggish reaction kinetics [18–20]. Thus, different measures including modulating morphology [21], constructing composites with conductive skeletons [22, 23] and introducing heterogeneous atoms were usually employed [24–26] to break through the bottleneck. Despite the improvement to some extent, these fabricated products still cannot meet the requirements for the high-performance ZIBs cathode materials, which greatly hinders the commercial applications of MnO_2_ in ZIBs field. Therefore, it is urgently needed to exploit a reliable strategy for realizing the target.

Vacancy engineering has become an effective strategy to boost the electrochemical performances of the transition metal oxides (TMOs) [27, 28]. It is mainly ascribed to the fact that the oxygen vacancies can act as shallow donors to redistribute the local charge states of the TMOs and significantly modulate the band gap and electron density, thereby fundamentally improving the conductivity [29, 30]. Meanwhile, the created vacancy greatly reduces the reaction energy barrier between the electrolyte and the active material, effectively increasing the reaction rate and accelerating the charge transfer [31]. Furthermore, the presence of vacancies in the lattice not only decreases the electrostatic repulsion between adjacent layers, greatly accelerating ion migration and reducing the stress induced by ions extraction and insertion [32–34], but also alleviates the spatial potential resistance of ion intercalation and effectively lessens the ions diffusion energy barrier [35, 36]. The advantages contribute to drastically promoting their reaction/diffusion kinetics, further elevating the rate capability. On the other hand, the introduction of oxygen vacancies can also generate more electrochemically active sites, increase the surface energy of the system and regulate the geometry of the TMOs, which helps the electrolyte ions to contact with more active materials, further resulting in more redox reactions and essentially increasing the charge storage capacity of the TMOs [37–39]. Recently, MnO_2_ with oxygen vacancies has been usually synthesized as the advanced electrode materials for rechargeable sodium-ion batteries and supercapacitors, for example, Chae et al. [40] used a simple solid-state reaction method to synthesize Ca_0.07_Na_0.26_MnO_2_ (CNMO) with many vacancy defects as advanced sodium-ion battery cathode materials, which can present high-rate capabilities and superior cycling stability (98.8% capacity retention after the 1000th cycle). Fu et al. [41] designed and constructed MnO_2_ with rich oxygen vacancies via a facile three-step method, which can be acted as cathode materials for supercapacitors. The obtained products can present superior specific capacitance of 452.4 F g^−1^ at 1 A g^−1^ and 316.1 F g^−1^ at 50 A g^−1^, respectively, which is better than that of the pure MnO_2_ (~ 240 F g^−1^ at 1 A g^−1^ and ~ 180 F g^−1^ at 30  A g^−1^, respectively). According to the above results, these oxygen vacancies can really enhance charge storage ability of these synthesized MnO_2_ electrode materials. Thus, numerous oxygen vacancies are introduced into the layered MnO_2_ and effectively modulate the vacancy concentration that can possibly present excellent electrochemical performances for ZIBs. To our knowledge, there are few reports in the previous study [42, 43] on the effective regulation of oxygen vacancies in manganese dioxide as a cathode for ZIBs with high specific capacity and rate capability.

In this paper, we used the hydrothermal and reduction treatments to construct typical flower-like *δ*-MnO_2_ nanostructures with optimal oxygen vacancies (*δ*-MnO_2−*x*_−2.0), which can be considered as an extraordinary cathode material for ZIBs. The unique cathode materials can hold a high specific capacity, significant rate capability and excellent cycle lifespan since the moderate vacancy level can ensure the ion chemisorption–desorption equilibrium and charge transfer rate during the reaction processes, as well as grant sufficient active sites, which is completely identified by the theoretical calculation. In addition, a co-insertion of H^+^/Zn^2+^ and phase transition charge storage mechanism in *δ*-MnO_2_ cathode are illustrated by the ex situ characterization techniques. This work presents a comprehensive understanding for the effect of oxygen vacancy modulation on the electrochemical performances of ZIBs cathode materials, further achieving a valuable design strategy for developing high-performance cathodes for ZIBs, which greatly facilitates the practical application prospects of aqueous ZIBs.

## Experimental Section

### Materials Preparation

A total of 50 mL of MnSO_4_ solution (0.025 mol L^−l^) were dissolved in 50 mL of KMnO_4_ solution (0.15 mol L^−l^) and fully mixed (the molar ratio of MnSO_4_ to KMnO_4_ is 1:1) through magnetic stirring. The mixed solution was magnetically stirred for 1 h and then transferred into a high-pressure reaction autoclave. Then, a piece of graphite substrate with a diameter of 12 mm was also placed in the above autoclave containing a mixed solution. Subsequently, the autoclave was placed into an oven and kept at 160 °C for 12 h. After it was cooled to room temperature, the graphite substrate deposited with some Sepia products are washed three times through deionized water and ethanol to obtain *δ*-MnO_2_. Finally, the as-prepared *δ*-MnO_2_ is treated by reduction treatment in KBH_4_ solution at room temperature, which can introduce rich oxygen vacancies into the *δ*-MnO_2_. Through controlling the reduction treatment time, the *δ*-MnO_2_ with various vacancy concentrations (*δ*-MnO_2−*x*_) can be obtained. Specifically, when the reduction treatment time is determined to be 0.5, 2 and 5 min, the as-prepared samples are named by *δ*-MnO_2−*x*_−0.5, *δ*-MnO_2−*x*_−2.0 and *δ*-MnO_2−*x*_−5.0. The obtained samples can directly act as the working electrodes.

### Materials Characterizations

FESEM (Hitachi, SU8010) and TEM (Hitachi, H-8100) were employed to record the morphology and microstructural information of the prepared samples. XRD (D8 X-ray diffractometer) was used to probe the phase compositions. XPS technique was carried out to further detect the surface composition and valence change on a Thermo ESCALAB 250Xi device with an Al-Kα (hν = 1486.6 eV) excitation source. In order to verify the introduction of the vacancies, electronic paramagnetic resonance (EPR) was conducted using a JES-FA200 EPR spectrometer at X-band (~ 9.4 GHz) with a resolution of 2.44 μT at room temperature. In addition, X-ray absorption fine structure spectroscopy (XAFS) was carried out at the beamline 1W1B of Beijing Synchrotron Radiation Facility. The electron beam energy of the storage ring was 2.5 GeV with ~ 250 mA.

### Electrochemical Characterizations

CR2025-type ZIB cells were constructed using the *δ*-MnO_2_ as the cathodes, zinc foils (10 µm) as the anodes and glass fiber paper as the separator. The aqueous mixture solution of 2.0 M ZnSO_4_ and 0.1 M MnSO_4_ was used as the electrolyte. Electrochemical workstation was employed to obtain cyclic voltammetry (CV) curves and electrochemical impedance spectrum (EIS). Galvanostatic charge/discharge technique and galvanostatic intermittent titration technique (GITT) were performed on Neware battery testing system to evaluate the specific capacity, rate capability, cycling stability and ion diffusion coefficient.

### Computational Details

The first-principle calculations invoke the Perdew–Burke–Ernzerhof (PBE) formulation of generalized gradient approximation (GGA) [44], and the projected augmented wave (PAW) potentials [45] were carried out via the Cambridge Sequential Total Energy Package (CASTEP) [46]. The GGA + U method is used to accurately describe the *d* electrons of transition metal Mn, and the Hubbard U parameter was set to be 2.0 eV. The typically exposed MnO_2_ (002) surface was modeled, whereas a vacuum spacing of 15 Å was employed to separate the periodic images. The kinetic energy cutoff was set as 300 eV, and a Monkhorst–Pack k mesh of 1 × 1 × 1 was adopted to sample the Brillouin zone. All atomic positions were fully relaxed until the forces are below 0.05 eV Å^−1^, and the electronic energy was considered self-consistent when the energy change was smaller than 1.0 × 10^–5^ eV per atom.

Moreover, ΔE is defined as Eq. (1):

1 $$\Delta {\text{E}} = {\text{E}}_{{{\text{ad}}/{\text{sub}}}} - {\text{E}}_{{{\text{ad}}}} - {\text{E}}_{{{\text{sub}}}}$$

in which E_ad/sub_, E_ad_ and E_sub_ are the total energies of the optimized adsorbate/substrate system, the adsorbate in the structure and the clean substrate, respectively.

Meanwhile, the free energy (ΔG) of H^+^ and Zn^2+^ on the pristine *δ*-MnO_2_ and *δ*-MnO_2−*x*_ surface was calculated based on Eq. (2):

2 $$\Delta {\text{G}} = \Delta {\text{E}} + {\text{ZPE}}{-}{\text{TS}}$$

where ΔE is the change of total energy from DFT calculations, ZPE and TS stand for the change of zero-point energy and the change of entropic contributions, respectively.

## Results and Discussion

### Structure, Phase, Valence State and Local Coordination Environment Characterization

**Scheme 1:**
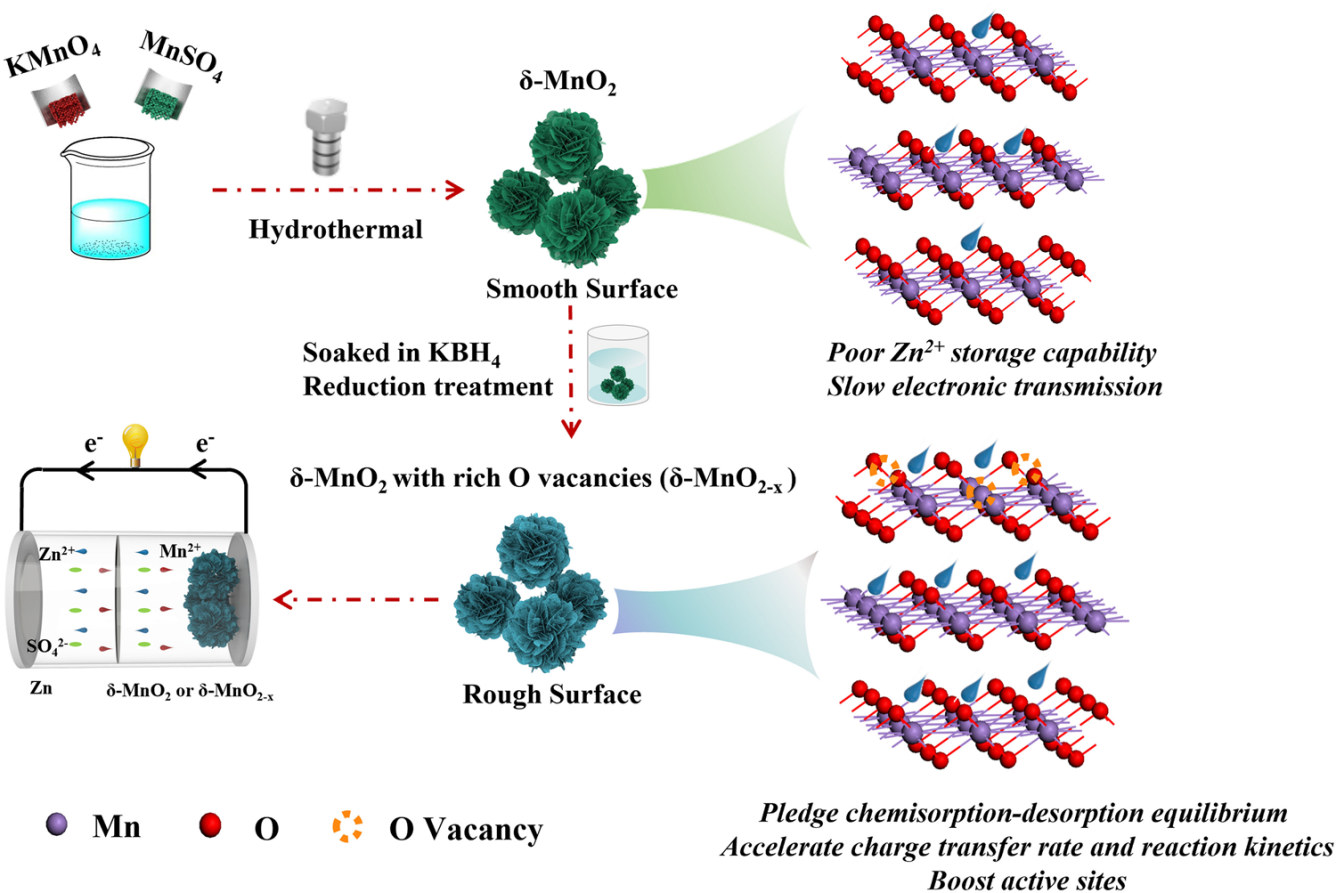
Schematic diagram of the preparation procedure for *δ*-MnO_2_ with rich oxygen vacancies (*δ*-MnO_2−*x*_) and the construction of a ZIB device

A schematic diagram of the synthesis process for *δ*-MnO_2−*x*_−2.0 and the construction of a ZIB device are shown in Scheme 1. The flower-like *δ*-MnO_2−*x*_−2.0 nanospheres are prepared via a simple hydrothermal method combined with a reduction treatment. First of all, potassium permanganate (KMnO_4_) and manganese sulfate (MnSO_4_), respectively, release ${\text{MnO}}_{4}^{ - }$ and Mn^2+^ in aqueous solution, then the obtained ${\text{MnO}}_{4}^{ - }$ can react with the above Mn^2+^ under high temperature to generate the *δ*-MnO_2_. The obtained *δ*-MnO_2_ present unique flower-like nanosphere structures assembled by a large number of smooth and interconnected nanosheets. Finally, numerous oxygen vacancies are introduced into the as-prepared *δ*-MnO_2_ through the reduction treatment in 0.1 M KBH_4_ solution at room temperature for 2 min to achieve *δ*-MnO_2−*x*_−2.0 products. After reduction treatment, the surface of these nanosheets becomes relatively rough and generates many pits at their edges, which can show more active sites to contact the electrolyte ions, thus maximizing the use of the products. Also, the obtained distinct structure effectively promotes charge transfer rate, H^+^/Zn^2+^ reaction kinetics and ensures the ions chemisorption–desorption equilibrium, which plays a vital role in enhancing their electrochemical performance. Furthermore, a ZIB device is successfully assembled with the fabricated *δ*-MnO_2−*x*_−2.0 and Zn foil as the cathode and anode, respectively, and *δ*-MnO_2−*x*_−0.5 and *δ*-MnO_2−*x*_−5.0 samples were also prepared by controlling reduction treatment time of 0.5 and 5 min.

**Fig. 1:**
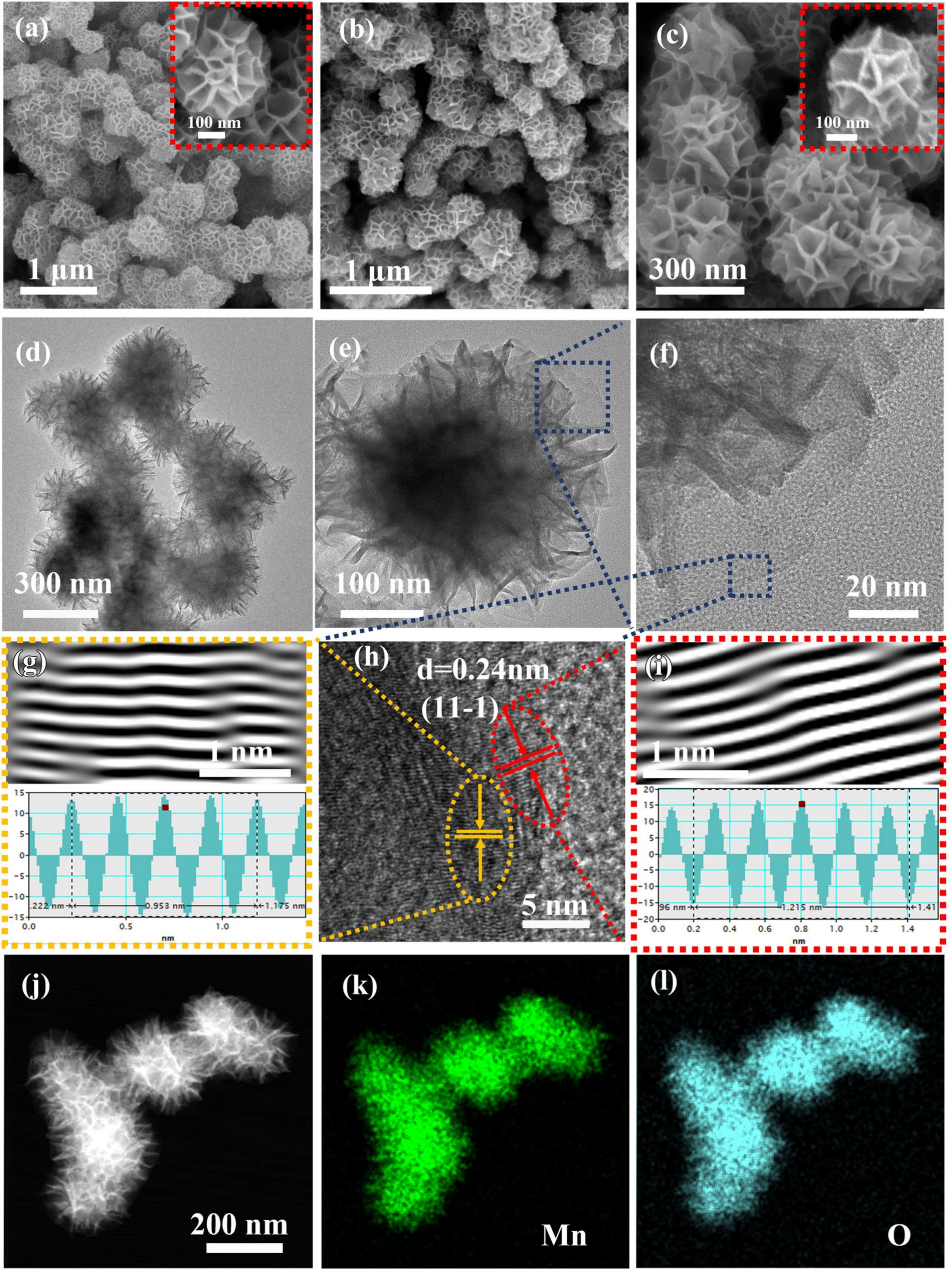
**a** SEM image of *δ*-MnO_2_ with low and high magnification, **b** low-magnification and **c** high-magnification SEM images of *δ*-MnO_2−*x*_−2.0, **d**–**f** TEM images and **g**–**i** HRTEM images of *δ*-MnO_2−*x*_−2.0 and **j**–**l** the corresponding EDS mapping of *δ*-MnO_2−*x*_−2.0

From scanning electron microscope (SEM) image of *δ*-MnO_2_ (Fig. 1a), it exhibits a typical flower-like nanosphere structures consisting of the smooth and intercrossed nanosheets. After introducing the moderate oxygen vacancies into the synthesized *δ*-MnO_2_, the micro-morphology of the obtained *δ*-MnO_2−*x*_−2.0 is similar to the *δ*-MnO_2_; however, the surface of these nanosheets becomes relatively rough because many pits exist on their edges (Fig. 1b and c). The distinct architecture can preserve the free open interspaces between the adjacent nanosheets, which can offer various ion transmission channels, effectively quickening the ions migration and guaranteeing the rapid ion diffusion kinetics. Moreover, the structure also enormously alleviates the possible volume change of the active materials induced by the ions intercalation/deintercalation during the charging/discharging process. Besides, the rough surface with numerous pits at their edges can provide adequate active sites for more redox reactions, which is in favor of the sufficient utilization of the active materials. Based on the aforementioned advantages, it can be expected to obtain the high-rate capability and large specific capacity. In addition, the SEM images of the control samples (*δ*-MnO_2−*x*_−0.5 and *δ*-MnO_2−*x*_−5) are depicted in Fig. S1. It can be seen from Fig. S1a, *δ*-MnO_2−*x*_−0.5 shows the well-defined flower-like structures, without any apparent difference in morphology compared with the *δ*-MnO_2−*x*_−2.0 (Fig. 1b and c). Nevertheless, the SEM image of *δ*-MnO_2−*x*_−5.0 shown in Fig. S1b illustrates that the nanosheets are seriously damaged, resulting in the collapse of the flower-like structures due to the excessive reduction treatment. Thus, the *δ*-MnO_2−*x*_−2.0 can be acted as the optimal sample, which will be discussed detailly in the following section. To further confirm the unique structure of the *δ*-MnO_2−*x*_−2.0, transmission electron microscope (TEM) technique is employed as presented in Fig. 1d–f. Clearly, the flower-like nanosphere structure of *δ*-MnO_2−*x*_−2.0 assembled in nanosheets, and the nanosheets are interconnected with each other, generating many free spaces, which is consistent with the SEM observations. The corresponding high-resolution transmission electron microscope (HRTEM) images shown in Fig. 1g–i display that the interlayer spacing of 0.24 nm (marked by red and yellow areas) all can assign to the (11–1) crystal plane of MnO_2_. In addition, elemental mapping of the X-ray energy-dispersive spectrum (EDS mapping) of the representative *δ*-MnO_2−*x*_−2.0 flower-like nanospheres (Fig. 1j–l) shows a uniform distribution of Mn and O across the surface of the products. Therefore, the above SEM and TEM analyses can provide ample evidence for the formation of the unique flower-like micro-structures of the *δ*-MnO_2−*x*_−2.0.

**Fig. 2:**
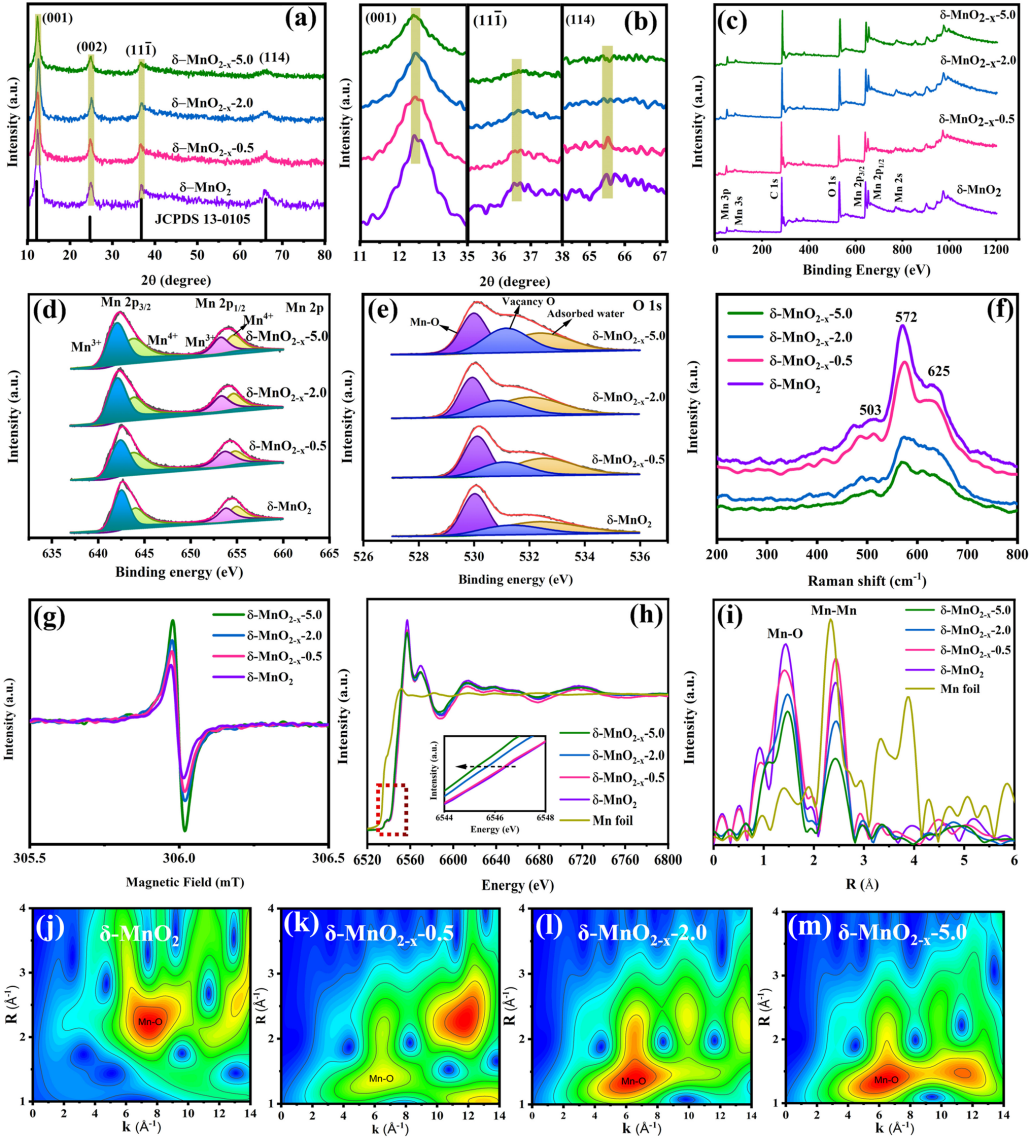
**a, b** XRD patterns of the *δ*-MnO_2_, *δ*-MnO_2−*x*_−0.5, *δ*-MnO_2−*x*_−2.0 and *δ*-MnO_2−*x*_−5.0. **c-e** XPS plots of *δ*-MnO_2_, *δ*-MnO_2−*x*_−0.5, *δ*-MnO_2−*x*_−2.0 and *δ*-MnO_2−*x*_−5.0, **a** the survey spectra and high-resolution spectra of **d** Mn 2p and **e** O 1*s*. **f** Raman spectra of the *δ*-MnO_2_, *δ*-MnO_2−*x*_−0.5, *δ*-MnO_2−*x*_−2.0 and *δ*-MnO_2−*x*_−5.0. **g** EPR of the *δ*-MnO_2_, *δ*-MnO_2−*x*_−0.5, *δ*-MnO_2−*x*_−2.0 and *δ*-MnO_2−*x*_−5.0. **h** XANES spectra, **i** the corresponding FT-EXAFS plots and **j**–**m** WT-EXAFS plots at Mn K-edge of *δ*-MnO_2_, *δ*-MnO_2−*x*_−0.5, *δ*-MnO_2−*x*_−2.0, *δ*-MnO_2−*x*_−5.0 and Ni foil

In order to examine the phase structure, valence and vacancy defect state of the prepared samples, X-ray diffraction (XRD), X-ray photoelectron spectroscopy (XPS), Raman spectra (Raman), electron paramagnetic resonance (EPR) and X-ray absorption fine structure (XAFS) techniques were employed, and the characterization results are shown in Fig. 2. From Fig. 2a and b, the main diffraction peaks located at ~ 12.2°, 24.7°, 36.8° and 66.2° correspond to (001), (002), (11–1) and (114) crystal planes, respectively, agreeing well with the *δ*-MnO_2_ (JCPDS No. 13–0105), and no obvious impurities are found. Notably, with the increase in oxygen vacancies, the diffraction peaks intensity of (001), (11–1) and (114) crystal planes of the *δ*-MnO_2−*x*_−0.5, *δ*-MnO_2−*x*_−2.0 and *δ*-MnO_2−*x*_−5.0 gradually become wider and weaker compared with the *δ*-MnO_2_, indicating that the created vacancy defects cause a typical disordered structures [47, 48]. Furthermore, it can be observed from Fig. 2c–e that the chemical composition and valence states of the above products were further investigated by XPS characterization. Figure 2c shows the survey spectra of the four samples, which demonstrates the presence of the Mn and O elements. In the Mn 2*p* XPS spectrum (Fig. 2d), a pair of peaks at 642.5 and 654.8 eV belong to Mn 2*p*_3/2_ and Mn 2*p*_1/2_ with a spin energy difference of 12.3 eV, confirming the characteristics of the manganese dioxide phase [49]. The four fitted peaks recorded from the Mn 2*p* suggest that Mn^4+^ and Mn^3+^ coexist in *δ*-MnO_2_. Interestingly, the Mn^3+^/Mn^4+^ integral area proportion gradually enhances from ~ 1.2 (*δ*-MnO_2_) to ~ 1.5 (*δ*-MnO_2−*x*_−5.0) because the conversion of Mn^4+^ to Mn^3+^ balances the introduced oxygen vacancies. The O 1*s* XPS spectra of the samples are depicted in Fig. 2e. The peaks located at 530.05, 531.20 and 532.55 eV are attributed to the Mn–O bonds, oxygen vacancy and adsorbed oxygen, respectively [50]. Obviously, *δ*-MnO_2−*x*_−5.0 presents larger oxygen vacancy peak intensity than that of other samples, further affirming evidently improved oxygen vacancy concentration after the reduction treatment, well consistent with the previously reported studies [51, 52]. Meanwhile, these samples were also investigated by Raman spectra analysis as displayed in Fig. 2f. It illustrates that the characteristic peaks at 503, 572 and 625 cm^−1^ are assigned to the stretching mode (Mn–O) of the MnO_6_ octahedron [53]. With the gradual reduction or even disappearance of these peaks in *δ*-MnO_2−*x*_−0.5, *δ*-MnO_2−*x*_−2.0 and *δ*-MnO_2−*x*_−5.0, it is strongly related that the effective change to the interlayer covalent interaction of Mn–O occurred after creating various oxygen vacancies [54]. Also, EPR measurements were carried out to provide fingerprint evidence for exploring the vacancies in the samples. As shown in Fig. 2g, they all possess an electron spin resonance (ESR) signal at *g* = 2.0 due to the electrons trapped on the vacancies [55]. The *δ*-MnO_2−*x*_−5.0 holds the highest ESR intensity among these samples, suggesting the existence of sufficient oxygen vacancies in the products. In addition, the presence of oxygen vacancies was further substantiated using XAFS spectroscopy. In the K-edge X-ray absorption near-edge structure (XANES) spectra of Mn (Fig. 2h), the Mn K-edge binding energy of the obtained samples gradually migrates to a lower direction as the augment of vacancy concentration, which reveals that the structural symmetry in *δ*-MnO_2_ is partially disturbed, and some Mn^4+^ are reduced to Mn^3+^ after forming oxygen vacancies into the products, which is in accordance with the XPS tests [56]. To better understand the coordination environment of the four samples, the Fourier transform extended X-ray absorption fine structure (FT-EXAFS) spectra of the Mn K-edge of *δ*-MnO_2_, *δ*-MnO_2−*x*_−0.5, *δ*-MnO_2−*x*_−2.0 and *δ*-MnO_2−*x*_−5.0 are conducted plotted in Fig. 2i. The strong peaks centered at ~ 2.2 and 2.5 Å in the Mn K-edge spectra infer to the Mn–O and Mn–Mn coordination states, respectively [57]. Clearly, the peak intensity of Mn–O for the *δ*-MnO_2_ is relatively higher than those of other samples. When the oxygen vacancies were created into the products, the corresponding peak intensity of Mn–O is gradually decreased ranging from *δ*-MnO_2−*x*_−0.5 to *δ*-MnO_2−*x*_−5.0, which can be ascribed to the removal of more oxygen sites, greatly increasing the degree of structural distortion [58]. As an important complement to FT-EXAFS, the visual wavelet transform (WT) contour maps own power resolution in R-space. As a result, the corresponding coordination path of the prepared samples can be obviously seen from the magnified WT images (Fig. 2j–m). All the above analyze results demonstrate that the controlled oxygen vacancies can be successfully introduced into the obtained samples by modulating reduction treatment time.

### Electrochemical Performances and Kinetics Analysis

**Fig. 3:**
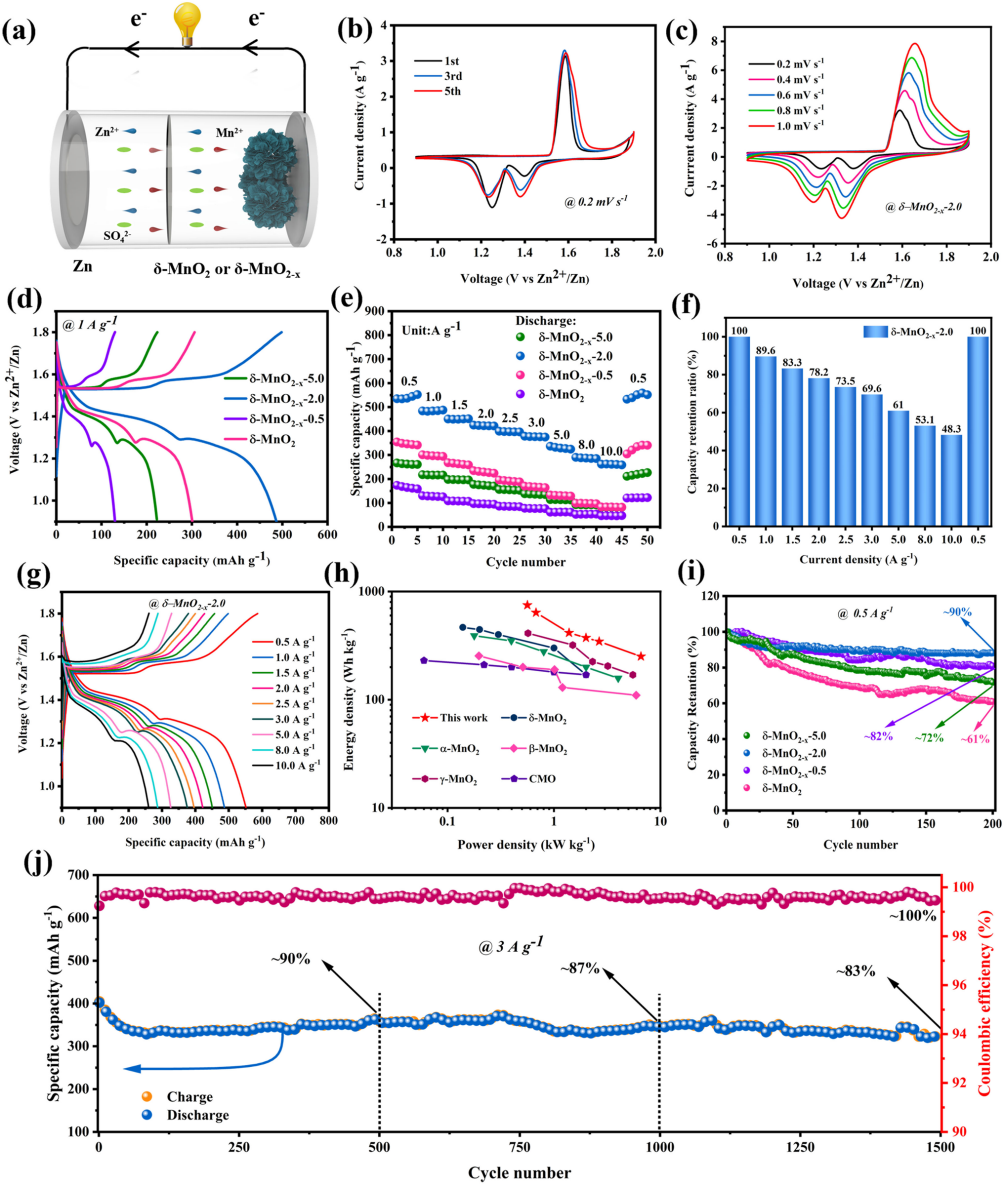
**a** Electrochemical performances of the *δ*-MnO_2_, *δ*-MnO_2−*x*_−0.5, *δ*-MnO_2−*x*_−2.0 and *δ*-MnO_2−*x*_−5.0 electrodes in ZIBs. Configuration of the aqueous ZIB with Zn foil as anode and the as-prepared *δ*-MnO_2_ or *δ*-MnO_2−*x*_ as cathode. **b** CV curves of *δ*-MnO_2−*x*_−2.0 at 0.2 mV s^−1^ for the initial five cycles. **c** CV curves of *δ*-MnO_2−*x*_−2.0 at 0.2 ~ 1.0 mV s^−1^. **d** Galvanostatic charge/discharge (GCD) plots of these obtained cathodes at 1.0 A g^−1^. **e** Rate performance. **f** Capacity retention rate of *δ*-MnO_2−*x*_−2.0. **g** GCD curves of *δ*-MnO_2−*x*_−2.0 at different current densities. **h** Ragone plots of *δ*-MnO_2−*x*_−2.0-based aqueous ZIBs with other types of cathode materials. **i**, **j** The cycling stability and coulombic efficiency at various current densities

To compare the electrochemical performances of *δ*-MnO_2_, *δ*-MnO_2−*x*_−0.5, *δ*-MnO_2−*x*_−2.0 and *δ*-MnO_2−*x*_−5.0 electrodes, the coin-type ZIBs were fabricated via employing Zn foil as anode, *δ*-MnO_2_ or *δ*-MnO_2−*x*_ as cathode and 2.0 M ZnSO_4_/0.1 M MnSO_4_ mixed solution as electrolyte, as shown in Fig. 3a. Based on the previous reports [59], the dissolution of Mn^2+^ can be effectively hindered by adding sufficient Mn^2+^ into the electrolyte during the charging process since it contributes to the formation of MnO_2_, significantly improving the effectiveness of the Zn plating and stripping. Thus, the electrolyte configuration plays a crucial role in promoting the electrochemical properties of the energy storage system [60]. Electrochemical properties of these obtained cathodes were achieved by CV tests in the voltage range of 0.9–1.9 V (vs. Zn^2+^/Zn) as shown in Figs. 3b–d and S2. Figure 3b presents the CV curves of *δ*-MnO_2−*x*_−2.0 from the first to the third cycles taken at 0.6 mV s^−1^. The two pairs of redox peaks suggest that the ZIB has experienced a two-step electrochemical reaction that refers to the insertion/extraction of H^+^ and Zn^2+^ ions. The CV curve area is continuously enlarged as the cycle continues for the initial five cycles, revealing the gradual activation process of the electrode materials [61]. Then, the excellent coincidence of the CV profile shapes in subsequent cycles illustrates that the redox reaction holds prominent reversibility and stability. Meanwhile, the CV measurements of the *δ*-MnO_2−*x*_−2.0 at various scan rates of 0.2–1.0 mV s^−1^ (Fig. 3c) were also conducted, which presents that all curves have a similar shape with two major pairs of redox peaks, and there is no distinct peak shift even at the high scan rate, indicating the favorable electronic/ionic conductivity. Moreover, galvanostatic charge/discharge (GCD) curves of *δ*-MnO_2_ and *δ*-MnO_2−*x*_ at the same current density of 1 A g^−1^ are compared in Fig. 3d, which clearly display that there are two discharge plateaus in the GCD curves located at ~ 1.59 and 1.29 V, respectively. According to the previous literature of MnO_2_-based aqueous ZIBs [62], the initial discharge platform corresponds to H^+^ insertion, while the later one is ascribed to Zn^2+^ insertion, which is almost in agreement with the oxidation and reduction peaks in CV plots. Among these obtained cathodes, the *δ*-MnO_2−*x*_−2.0 can deliver superior electrochemical activity and larger reversible capacity of 551.8 mAh g^−1^ than those of the other cathodes (173.5, 348.5 and 266.8 mAh g^−1^). Rate capability as an essential indicator for the realistic application of *δ*-MnO_2_ and *δ*-MnO_2−*x*_ in aqueous ZIBs is assessed at 0.5 ~ 10.0 A g^−1^ (Figs. 3e-g and S3). From Figs. 3e, f and S4, when the current density is 0.5, 1.0, 1.5, 2.0, 2.5, 3.0 and 5.0 A g^−1^, respectively, the specific capacities of 551.8, 487.1, 451.0, 420.7, 396.3, 375.4 and 328.6 mAh g^−1^ are achieved for *δ*-MnO_2−*x*_−2.0 cathode. As the current density increases to 10.0 A g^−1^, it can still hold a reversible capacity of 262.2 mAh g^−1^ with 48% capacity retention, substantiating the outstanding rate performance of *δ*-MnO_2−*x*_−2.0 cathode. In contrast, the pristine *δ*-MnO_2_, *δ*-MnO_2_−0.5 and *δ*-MnO_2_−5.0 cathodes only present a lower capacity retention rate of 23.9%, 28.2% and 31.1% at 10.0 A g^−1^, respectively. Also, the corresponding GCD curves of these cathodes at various current densities further verify the best electrochemical performances of *δ*-MnO_2−*x*_−2.0 among the as-prepared cathodes (Figs. 3g and S4). The performance comparison of the *δ*-MnO_2−*x*_−2.0 with different reported transition metal compound cathodes in aqueous ZIBs is shown in Table S1. Obviously, the *δ*-MnO_2−*x*_−2.0 presents larger capacity and higher rate capability than those of the other products, which may be benefited from the enhanced electronic conductivity and ion chemisorption–desorption equilibrium, as well as the sufficient electroactive sites owing to the effective modulation of the oxygen vacancies in the *δ*-MnO_2_. The Ragone plots in Fig. 3g clearly suggest that the energy density of *δ*-MnO_2−*x*_−2.0 is 747.5 Wh kg^−1^ at a power density of 561.6 W kg^−1^, and its energy density can still maintain 373.5 Wh kg^−1^ when the power density is increased to 2000 W kg^−1^, which outperforms other ZIB cathodes such as *δ*-MnO_2_ [63], *α*-MnO_2_ [59], *β*-MnO_2_ [64], *γ*-MnO_2_ [65] and CMO [66]. In addition, the obtained *δ*-MnO_2−*x*_−2.0 cathode can display excellent cycling stability as shown in Fig. 3i and j. Specifically, the capacity retention of *δ*-MnO_2−*x*_−2.0 is ~ 90% after 200 continuous charge/discharge cycles at 0.5 A g^−1^, which is superior than those of other prepared cathodes (~ 82%, ~ 61% and ~ 72% for *δ*-MnO_2_, *δ*-MnO_2−*x*_−0.5 and *δ*-MnO_2−*x*_−5.0, respectively). More significantly, the *δ*-MnO_2−*x*_−2.0 can still preserve ~ 83% capacity retention and ~ 100% coulombic efficiency after 1500 cycles even at a high current density of 3.0 A g^−1^, demonstrating the remarkable long-term cycling lifespan. Thus, the *δ*-MnO_2−*x*_−2.0 electrode possesses an excellent cycling stability, which is also clearly more favorable than those of the other battery-type electrodes in prior reports displayed in Table S2.

**Fig. 4:**
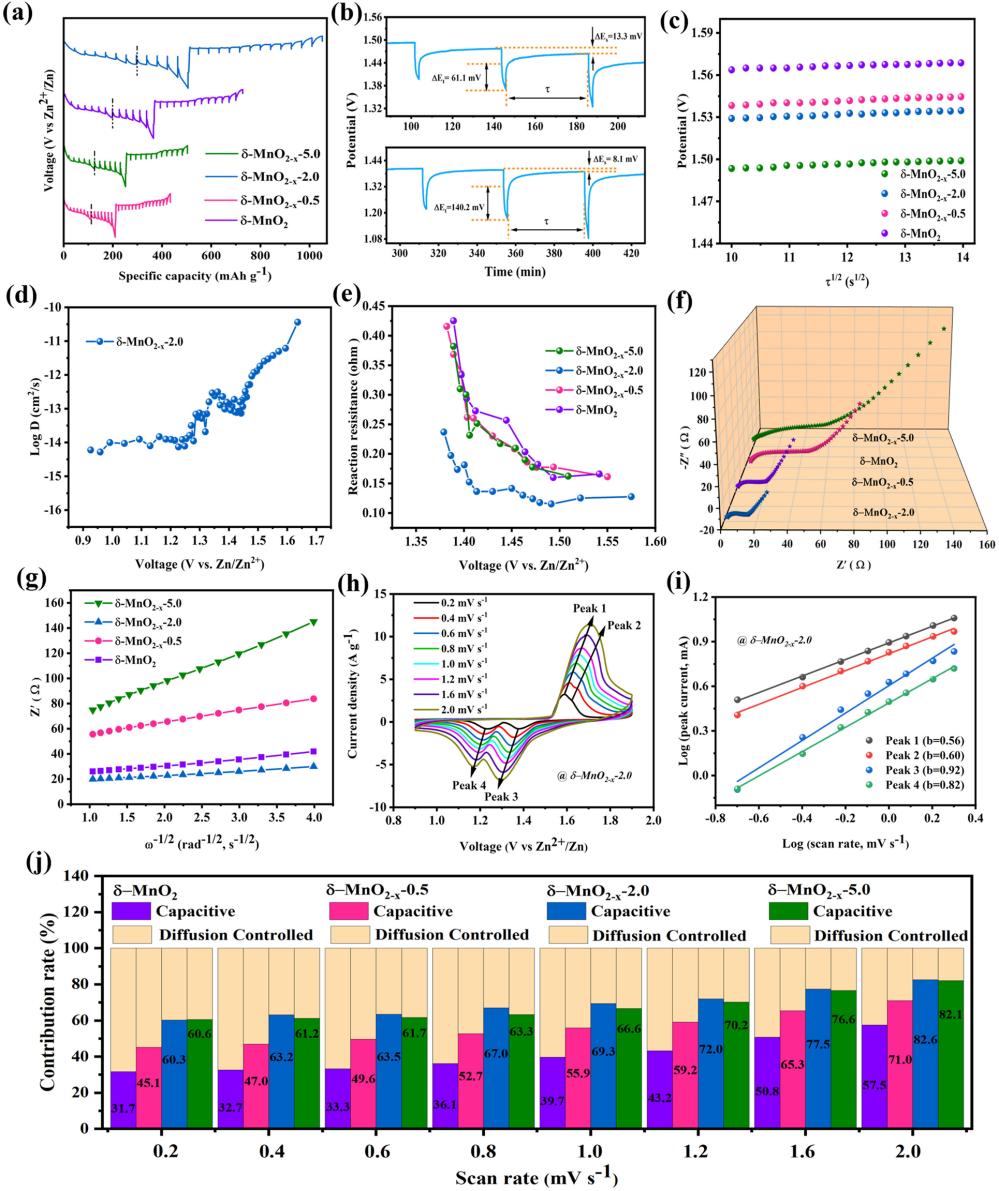
Kinetic analysis of the obtained *δ*-MnO_2_, *δ*-MnO_2−*x*_−0.5, *δ*-MnO_2−*x*_−2.0 and *δ*-MnO_2−*x*_−5.0 cathodes. **a** GITT profiles. **b** Titration plots with a schematic illustration of different parameters. **c** Variation of the potential against τ^1/2^. **d** In situ zinc-ion diffusion coefficient of *δ*-MnO_2−*x*_−2.0. **e** Reaction resistances of the *δ*-MnO_2_, *δ*-MnO_2−*x*_−0.5, *δ*-MnO_2−*x*_−2.0 and *δ*-MnO_2−*x*_−5.0, respectively. **f** EIS spectra and **g** the corresponding relationship between Z_real_ and ω^−1/2^ of the *δ*-MnO_2_, *δ*-MnO_2−*x*_−0.5, *δ*-MnO_2−*x*_−2.0 and *δ*-MnO_2−*x*_−5.0. **h** CV curves at different scan rates and **i** log i versus log v plots at four peaks of the *δ*-MnO_2−*x*_−2.0 cathode. **j** Contribution ratios of capacitive-like and diffusion-controlled capacities at different scan rates for the *δ*-MnO_2_, *δ*-MnO_2−*x*_−0.5, *δ*-MnO_2−*x*_−2.0 and *δ*-MnO_2−*x*_−5.0 cathodes

GITT and electrochemical impedance spectroscopy (EIS) and CV measurements were employed to probe the energy storage mechanism and kinetic behaviors, as displayed in Fig. 4. As for the *δ*-MnO_2−*x*_−2.0 cathode, the GITT profile the amplified titration curves with a schematic illustration of *Δ*Es, *Δ*Eτ and τ (in which *Δ*Es is the voltage change between steps, *Δ*Eτ and τ are the voltage change and the constant current pulse, respectively) show that the total overvoltage (13.3 mV) of the *δ*-MnO_2−*x*_−2.0 cathode in region II is about 1.64 times that of the value (8.1 mV) in region I, which is induced by a large voltage jump and sluggish ion diffusion in region II (Fig. 4a and b). According to the previous study [62], it is found that the first discharge platform corresponds to the intercalation of H^+^, while the second discharge platform is mainly assigned to the intercalation of Zn^2+^. The other obtained cathodes can also present similar phenomenon (Fig. 4a). Then, combined with the linear variation of the potential against τ^1/2^ (Fig. 4c) and Fick’s second law, the corresponding diffusion coefficients of the as-fabricated *δ*-MnO_2_, *δ*-MnO_2−*x*_−0.5, *δ*-MnO_2−*x*_−2.0 and *δ*-MnO_2−*x*_−5.0 cathodes can be ascertained. As can be seen from the calculated results (Fig. 4d), the diffusion coefficient of the *δ*-MnO_2−*x*_−2.0 in the first stage (10^–11^–10^–14^ cm^2^ s^–1^) is greatly larger than that of the second one (10^–14^–10^–15^ cm^2^ s^–1^). The apparently decreased diffusion coefficient of the second stage illustrates that the intercalated cation species are different. Given the difference in ionic radius size of H^+^ and Zn^2+^, the H^+^ intercalation with smaller radius size may refer to the first discharge plateaus, and the second platform should conduct Zn^2+^ insertion, further certifying the H^+^ and Zn^2+^ co-insertion in the *δ*-MnO_2−*x*_−2.0 cathode. Notably, the diffusion coefficient of the *δ*-MnO_2−*x*_−2.0 is significantly higher than those of the other obtained cathodes (Figs. 4d and S5), suggesting its more excellent kinetic behaviors, which is largely attributed to the vital role of the introduced moderate vacancies in facilitating ion diffusion. Moreover, during the electrochemical reaction, the in situ reaction resistance at various Zn^2+^ insertion/extraction stages can be calculated on the basis of the closed-circuit voltage (CCV) and quasi-open-circuit voltage (QOCV) of the GITT profiles [67]. As depicted in Fig. 4e, the reaction resistance of the *δ*-MnO_2−*x*_−2.0 is lowest among these cathodes, which effectively accelerates the charge transmission kinetics for efficient Zn^2+^ storage. Furthermore, the EIS spectra and the corresponding relationship between Z_real_ and ω^−1/2^, as well as the capacitive/diffusion-controlled contribution, are tested to achieve a deep understanding of the kinetic behaviors of *δ*-MnO_2_, *δ*-MnO_2−*x*_−0.5, *δ*-MnO_2−*x*_−2.0 and *δ*-MnO_2−*x*_−5.0 cathodes, as displayed in Fig. 4f–j. It can obviously find that the *δ*-MnO_2−*x*_−2.0 not only delivers the lower intrinsic resistance (R_s_) than those of the counterpart samples, but also presents a more satisfied straight line along the imaginary axis, illuminating a smaller diffusion resistance (R_w_) for effective ion transport (Fig. 4f). Besides, the relationship between Z_real_ and ω^−1/2^ of *δ*-MnO_2_, *δ*-MnO_2−*x*_−0.5, *δ*-MnO_2−*x*_−2.0 and *δ*-MnO_2−*x*_−5.0 is also determined in Fig. 4g based on Eq. (3) [68]:

3 $${\text{Z}}_{{{\text{real}}}} = {\text{R}}_{{\text{e}}} + {\text{R}}_{{{\text{ct}}}} + \sigma \omega^{{ - {1}/{2}}}$$

Theoretically, the smaller Warburg factor (σ) of the cathode, the rapider the ion diffusion rate. The σ of the *δ*-MnO_2−*x*_−2.0 is 3.3, which is much lower than those of *δ*-MnO_2_ (5.3), *δ*-MnO_2−*x*_−0.5 (9.5) and *δ*-MnO_2−*x*_−5.0 (23.2), effectively verifying that the *δ*-MnO_2−*x*_−2.0 possesses the faster ion diffusion rate. In addition, in light of the CV curves at different scan rates (Fig. 4h), the electrochemical kinetics source can be defined via Eq. (4) [69]:

4 $$i = {\text{a}}v^{{\text{b}}}$$

in which *i* (A) is the peak current, a and b are adjustable parameters and *v* (mV s^−1^) is the scan rate [70]. Based on the linear relation, the b values of the four peak currents for the *δ*-MnO_2−*x*_−2.0 can be calculated to be 0.61 (peak 1), 0.62 (peak 2), 0.82 (peak 3) and 0.65 (peak 4), respectively (Fig. 4i), indicating that the capacitive-controlled behavior and diffusion-limited contribution synergistically dominate the charge storage process [71, 72]. The obtained b values of the *δ*-MnO_2−*x*_−2.0 are larger than those of the *δ*-MnO_2_, *δ*-MnO_2−*x*_−0.5 and *δ*-MnO_2−*x*_−5.0 (Fig. S6), which reveals that the electrochemical reaction of the *δ*-MnO_2−*x*_−2.0 preserves more capacitive behavior compared with the other cathodes. The contributions of capacitance (k_1_*v*) and diffusion (k_2_*v*^1/2^) components are further quantified according to Eq. (5) [73]:

5 $$i = {\text{k}}_{{1}} v + {\text{k}}_{{2}} v^{{{1}/{2}}}$$

where *i* is the current at a fixed potential. The capacitive contribution ratios at various scan rates are achieved as displayed in Fig. 4j. Obviously, the capacitive contribution of the *δ*-MnO_2−*x*_−2.0 accounts for 60.3% of the total capacity at 0.2 mV s^−1^, and it gradually increases to 82.6% when the scan rate reaches 2.0 mV s^−1^, which is larger compared to the other cathodes, substantiating the faster electrochemical kinetics. The favorable capacitive behavior together with the rapid Zn^2+^ diffusivity is responsible for the satisfactory rate capability of the *δ*-MnO_2−*x*_−2.0 cathode.

### Theoretical Insight into the Optimum Performances

**Fig. 5:**
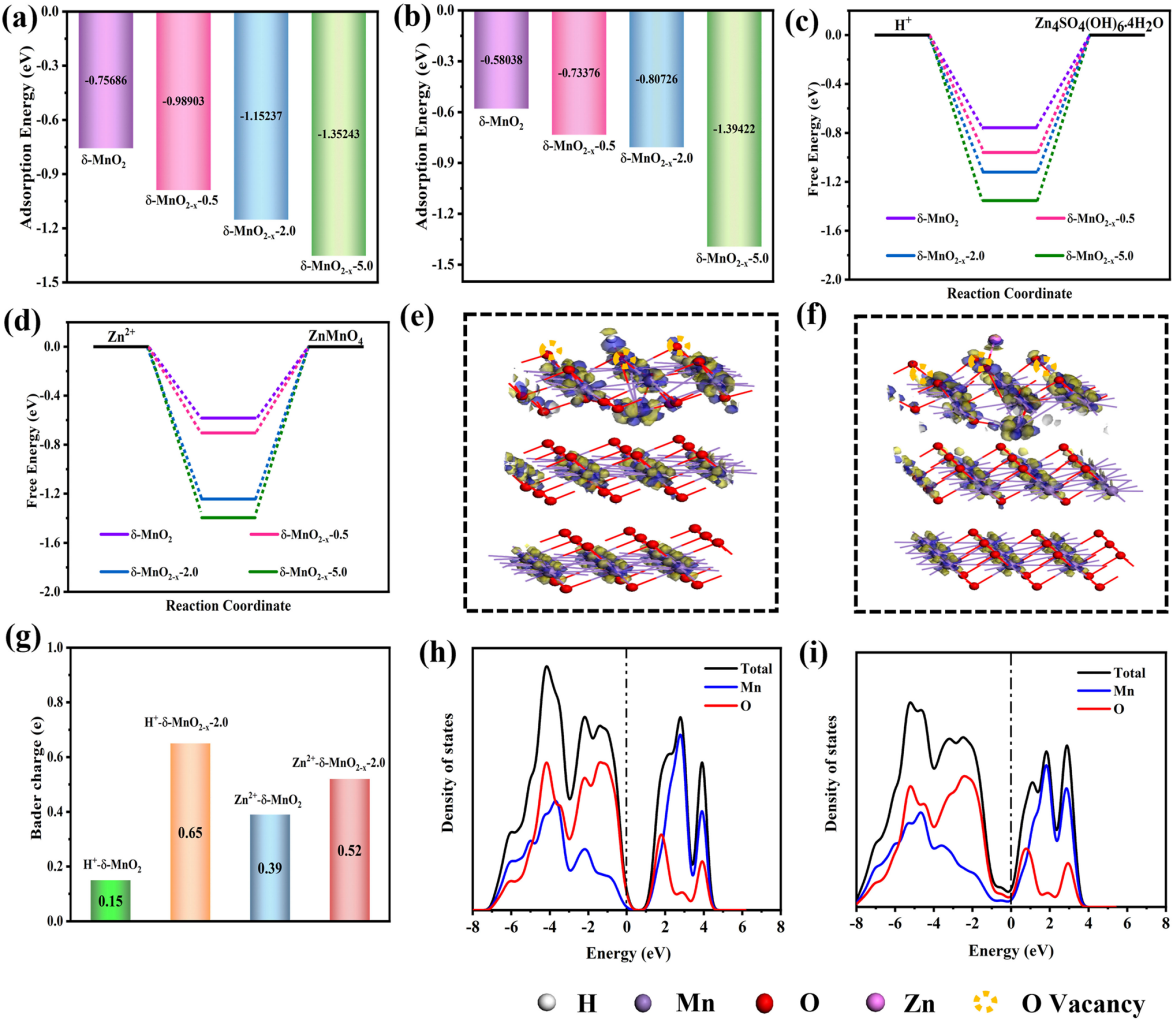
DFT calculations of the prepared cathode materials. The adsorption energies of **a** H^+^ and **b** Zn^2+^ on *δ*-MnO_2_, *δ*-MnO_2−*x*_−0.5, *δ*-MnO_2−*x*_−2.0 and *δ*-MnO_2−*x*_−5.0, respectively. **c**, **d** Gibbs free energy diagram of charging storage on these samples. The differential charge density maps of *δ*-MnO_2−*x*_−2.0 with **e** H^+^ and **f** Zn^2+^ adsorption and **g** the corresponding Bader charges, in which yellow and blue areas stand for the electron accumulation and depletion, respectively. The density of states (DOS) of the **h** *δ*-MnO_2_ and **i** *δ*-MnO_2−*x*_−2.0

**Fig. 6:**
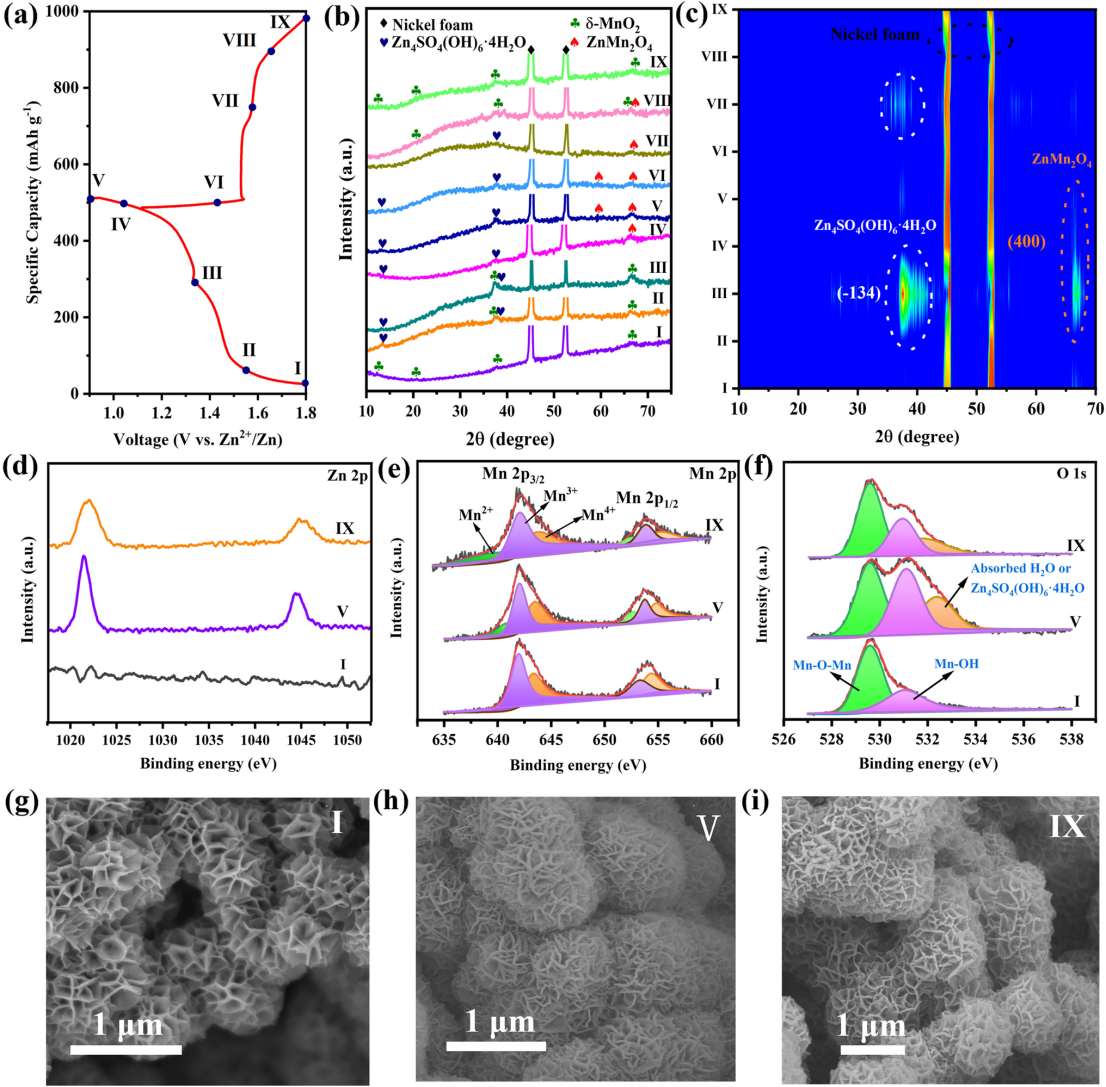
**a** Charge storage mechanism of *δ*-MnO_2−*x*_−2.0 cathode based on the *ex situ* characterization technique. GCD plots of the *δ*-MnO_2−*x*_−2.0 at different charge/discharge steps. **b**, **c** Ex situ XRD patterns of the *δ*-MnO_2−*x*_−2.0 at different charge/discharge states. Ex situ XPS high-resolution spectra of **d** Zn 2*p*, **e** Mn 2*p* and **f** O 1*s* of the *δ*-MnO_2−*x*_−2.0 at different charge/discharge stages. **g**–**i** Ex situ SEM images of the the *δ*-MnO_2−*x*_−2.0 during various electrochemical steps

To further deeply understand the positive effects of vacancy modulation on improving the electrochemical performances, density functional theory (DFT) calculations were performed, and the corresponding analysis results are shown in Fig. 5. The geometry configurations of the *δ*-MnO_2_, *δ*-MnO_2−*x*_−0.5, *δ*-MnO_2−*x*_−2.0 and *δ*-MnO_2−*x*_−5.0, as well as the adsorbed H^+^/Zn^2+^ at the above models, were optimized (Figs. S7–S8). The charge storage mechanism for the cathode materials can be unveiled through Eqs. (6–9) [74], which is also analyzed detailly in the following discussion (Fig. 6):

6 $${\text{H}}_{{2}} {\text{O}} \leftrightarrow {\text{H}}^{ + } {\text{ + OH}}^{ - }$$

7 $$\frac{{1}}{{2}}{\text{Zn}}^{{2 + }} {\text{ + OH}}^{ - } { + }\frac{{1}}{{6}}{\text{ZnSO}}_{{4}} { + }\frac{{2}}{{3}}{\text{H}}_{{2}} {\text{O}} \leftrightarrow \frac{{1}}{{6}}{\text{ZnSO}}_{{4}} {\text{[Zn(OH)}}_{{2}} {]}_{{3}} \cdot {\text{4H}}_{{2}} {\text{O}}$$

8 $${\text{MnO}}_{{2}} {\text{ + H}}^{ + } {\text{ + e}}^{ - } \leftrightarrow {\text{MnOOH}}$$

9 $${\text{Zn}}^{{2 + }} {\text{ + 2e}}^{ - } {\text{ + 2MnO}}_{{2}} \leftrightarrow {\text{ZnMn}}_{{2}} {\text{O}}_{{4}}$$

where H^+^/Zn^2+^ ions all involve the charge/discharge process. To illustrate the redox activity and charge storage reversibility, the adsorption energy of H^+^/Zn^2+^ (*Δ*E_H_^+^/ΔE_Zn_^2+^).

on the above cathodes and the Gibbs free energy (Δ*G*_H_^+^/Δ*G*_Zn_^2+^) of the redox reactionswere estimated, as shown in Fig. 5a–d. Specifically, Δ*E*_H_^+^ of *δ*-MnO_2_, *δ*-MnO_2−*x*_−0.5, *δ*-MnO_2−*x*_−2.0 and *δ*-MnO_2−*x*_−5.0 is −0.757, −0.989, −1.15 and −1.35 eV, respectively (Fig. 5a), and their ΔE_Zn_^2+^ is −0.58, −0.734, −0.807 and 1.39 eV (Fig. 5b). These data indicate that the H^+^/Zn^2+^ adsorption strength of *δ*-MnO_2_ with oxygen vacancies is significantly higher than that of bare *δ*-MnO_2_. With the increases in the oxygen vacancies created in *δ*-MnO_2_, the H^+^/Zn^2+^ adsorption strength is further enhanced. Thus, the H^+^/Zn^2+^ value of *δ*-MnO_2−*x*_−5.0 is the largest among these samples. A similar trend is observed for the Gibbs free energies of the Zn_4_SO_4_(OH)_6_.4H_2_O and ZnMn_2_O_4_ formation reaction, as displayed in Fig. 5c and d. Thus, for the discharging process (right arrows) expressed using formula 7, the vacancy engineering can elevate the reaction rate and specific capacity. Theoretically, the *δ*-MnO_2−*x*_−5.0 cathode possesses prominent electrochemical performances, whereas the *δ*-MnO_2−*x*_−2.0 cathode (corresponding Δ*G*_H_^+^ = −1.12 eV, Δ*G*_Zn_^2+^ = −1.24 eV) verifies the highest specific capacity and rate capability in practice. It is because extremely robust H^+^/Zn^2+^ binding strength referring to *δ*-MnO_2−*x*_−5.0 (Δ*G*_H_^+^  = −1.35 eV, Δ*G*_Zn_^2+^  = −1.40 eV) may give rise to more difficulties in completing the charge reaction (left arrows) than that of the *δ*-MnO_2−*x*_−2.0, bringing about immensely inferior reversibility in *δ*-MnO_2−*x*_−5.0. Accordingly, *δ*-MnO_2−*x*_−2.0 provides relatively strong and moderate H^+^/Zn^2+^ absorption capability compared to the other obtained cathode materials. Therefore, the *δ*-MnO_2−*x*_−2.0 displays optimal H^+^/Zn^2+^ chemisorption–desorption equilibrium, which makes the reversible reactions easier to proceed, effectively accelerating the charge transfer rate during the redox reaction process and further demonstrating outstanding rate capability. Meanwhile, it greatly enhances the reaction activities, substantially guaranteeing the high capacity. Based on the above results, the introduced oxygen vacancies in the cathode materials own an optimal level rather than an infinitely improving vacancy concentration. Thus, our study offers a unique path for determining the optimal vacancy concentration, which is of extreme significance for constructing high-performance ZIBs cathode materials. In order to explore the bonding properties of the adsorbed H^+^/Zn^2+^ in the optimal *δ*-MnO_2−*x*_−2.0 and *δ*-MnO_2_, the corresponding electron density difference was calculated presented in Figs. 5e, f and S8, respectively. Evidently, the charge difference maps of the adsorbed H^+^/Zn^2+^ at the *δ*-MnO_2−*x*_−2.0 in Fig. 5e and f present noticeable migration of valence electrons, wherein it holds larger accumulation/depletion area than that of the pristine *δ*-MnO_2_ (Fig. S8), which is quantitatively testified using the Bader charge analysis (the specific charge transfer level is 0.65/0.15 e and 0.52/0.39 e for *δ*-MnO_2−*x*_−2.0 and *δ*-MnO_2_, respectively, Fig. 5g). The above analysis indicates that more redox reactions between *δ*-MnO_2−*x*_−2.0 and H^+^/Zn^2+^ can occur owing to the moderate oxygen vacancy states, thereby rendering higher charge storage capacity. In addition, the density of states (DOS) of the *δ*-MnO_2−*x*_−2.0 and *δ*-MnO_2_ (Fig. 5h and i) obviously display a higher intensity near the Fermi level in *δ*-MnO_2−*x*_−2.0 compared with the *δ*-MnO_2_, which reveals that the *δ*-MnO_2−*x*_−2.0 can deliver more excellent electronic conductivity than that of the prepared *δ*-MnO_2_ cathode. The theoretical calculation is consistent with our experimental results, which fundamentally uncovers the reasons why the *δ*-MnO_2_ cathode with moderate oxygen vacancies can manifest high-rate property and large capacity.

### Charge Storage Mechanism of the ***δ***-MnO_2−*x*_−2.0

To further explore the energy storage mechanism of the *δ*-MnO_2−*x*_−2.0 cathode, a series of ex situ XRD, XPS and SEM measurements were carried out at the specific charged and discharged states based on the first GCD profiles presented in Fig. 6a. Figure 6b and c illustrates its ex situ XRD patterns at various states in the discharge/charge process, which clearly manifests that during the first discharge platform (point I), there are four major diffraction peaks of the *δ*-MnO_2_ located at 12.2°, 24.7°, 36.8° and 66.2° can be clearly observed, and the other two strong diffraction peaks centered at 45.4° and 52.6° are assigned to nickel foam substrate. Subsequently, some new peaks at 13.5° and 37.6° marked by the blue symbol resulted from the points II and III can match well with the zinc sulfate hydroxide hydrate (ZSH, Zn_4_SO_4_(OH)_6_.4H_2_O, JCPDS No. 44-0673) [75, 76]. The generation of ZSH is attributed to the enhanced amount of OH^–^along with theconsumption of H^+^, which is consistent well with the previous studies [77, 78]. Noted that no apparent peak of MnOOH is probed in the *ex* situ XRD patterns, presumably due to the H^+^ inserting into the interlayer of *δ*-MnO_2_ [79]. Except for the peaks of ZSH, the typical peaks of the ZnMn_2_O_4_ (ZMO, JCPDS No. 77–0470) phase labeled by red symbol at 59.1° and 66.3° can be detected during the discharge process (points IV and V) because of the Zn^2+^ intercalation. In the following charging process (points VI–IX), these new emerged diffraction peaks of the above produced ZSH and ZMO gradually vanish, and then, the intrinsic peaks of the *δ*-MnO_2_ can also recover well, indicating the superior reversibility of the *δ*-MnO_2−*x*_−2.0 cathode. Furthermore, the ex situ XPS tests were utilized to further demonstrate the phase transition mechanism during the discharge/charge process. In terms of the high-resolution Zn 2*p* spectra (Fig. 6d), no peaks are found in the initial state (I state), which suggests that the Zn element does not exist in the *δ*-MnO_2−*x*_−2.0. After discharging to V state, a pair of evident characteristic peak signals located at 1022.2 and 1044.8 eV are detected resulted from the insertion of Zn^2+^ into the *δ*-MnO_2−*x*_−2.0 or the generation of the ZSH products, but the peaks intensities are greatly decreased induced by most of the Zn^2+^ extraction from the cathode or the decomposition of ZSH after recharging to IX state. Figure 6e shows the high-resolution Mn 2*p* spectra at different stages. Compared with the pristine I state, a new peak discovered at 641.6 eV is ascribed to Mn^2+^, and the peaks intensities of Mn^3+^and Mn^4+^ obviously diminish at fully discharged V state, which further substantiates the reduction of Mn^3+^and Mn^4+^ caused by the Zn^2+^ insertion according to Eq. (10). When the cathode was charged to IX state again, the peak integral area of Mn^3+^and Mn^4+^ can be also significantly augmented owing to the extraction of Zn^2+^ from the cathode. Notably, the Mn^2+^ peak signal does not completely disappear, which can be mainly attributed to the captured Zn^2+^ in the cathode. Moreover, the O 2*p* spectra (Fig. 6f) show that an apparent peak located at 532.8 eV can be observed, which can correspond to the formed ZSH or absorbed H_2_O after discharging to V state, and the peak intensity enormously reduces at fully charged IX state because of the decomposition of ZSH and the extraction of H_2_O. In addition, the micro-morphology evolution of the *δ*-MnO_2−*x*_−2.0 cathode materials was further investigated using *ex situ* SEM characterization (Figs. 6g–i and S9). As for the initial state (I state), there is no obvious change of the flower-like morphology on the cathode materials surface. However, when it was discharged to V state, these nanosheets become thicker and rougher caused by the initially generated ZSH and ZMO products on their surface, and then almost completely disappeared at IX state. The highly reversible behaviors during the discharge/charge process accord well with the aforementioned ex situ XRD and XPS analysis results and the previously reported *ex situ* characterizations [80].

## Conclusions

In summary, oxygen vacancies modulation strategies have been successfully employed to construct the unique flower-like *δ*-MnO_2_ nanostructures with moderate oxygen vacancies as the advanced ZIB cathodes. The created moderate oxygen vacancies in *δ*-MnO_2_ (*δ*-MnO_2−*x*_−2.0) not only effectively ensures H^+^/Zn^2+^ chemisorption–desorption equilibrium, making the redox reactions easier to proceed and further accelerating the electron transfer rate during the reactions, but also intrinsically enhances electrochemical activity and electrical conductivity, leading to faster reaction kinetics and more reversible redox reactions, as demonstrated by the series of the test techniques and the corresponding DFT calculations. Benefiting from the above advantages, the prepared *δ*-MnO_2−*x*_−2.0//Zn ZIBs can deliver a maximum specific capacity of 551.8 mAh g^−1^ at 0.5 A g^−1^, ultrahigh rate capability (262.2 mAh g^−1^ even at 10 A g^−1^) and a long-term cycle lifespan with a capacity retention ~ 90% after 1500 cycles. Meanwhile, the assembled ZIBs can also present a large energy density of 373.5 Wh kg^−1^ at 2.0 kW kg^−1^, and when the power density is increased to 747.5 kW kg^−1^, it still possesses the favorable energy density of 561.6 Wh kg^−1^. This work both provides a potential cathode candidate for next-generation aqueous ZIBs and unfolds an effective vacancy modulation strategy for constructing the other high-performance battery electrode materials.

### Supplementary Information

Below is the link to the electronic supplementary material.

Supplementary file1 (PDF 1762 kb)
