# Supplementary material for: Effectively Modulating Oxygen Vacancies in Flower-Like δ-MnO2 Nanostructures for Large Capacity and High-Rate Zinc-Ion Storage
Source: Nanomicro Lett. 2023 Oct 7;15:219. doi: 10.1007/s40820-023-01194-3 (PMC10560176; doi:10.1007/s40820-023-01194-3)
Supplement: Supplementary file 1 — Supplementary file1 (PDF 1762 kb) [file 40820_2023_1194_MOESM1_ESM.pdf]

Supporting Information for

Effectively Modulating Oxygen Vacancies in Flower-Like δ-MnO_2 Nanostructures for Large Capacity and High-Rate Zinc Ion Storage

Yiwei Wang[1], Yuxiao Zhang[1], Ge Gao[1], Yawen Fan[1], Ruoxin Wang[1], Jie Feng[1], Lina Yang[2], Alan Meng[2], Jian Zhao[1,] *, Zhenjiang Li[1,] *

[1]College of Materials Science and Engineering, Qingdao University of Science and Technology, Qingdao 266042, Shandong, P. R. China

[2]College of Chemistry and Molecular Engineering, Qingdao University of Science and Technology, Qingdao 266042, Shandong, P. R. China

*Corresponding authors. E-mail: zjcc2016@qust.edu.cn (Jian Zhao)

zhenjiangli@qust.edu.cn or zjli126@126.com (Zhenjiang Li)

S1 Calculations

The corresponding diffusion coefficients of the δ-MnO_2 and δ-MnO_{2-x} electrodes are obtained according to the equation:

$$D_{GITT}=\frac{4}{\pi\tau}\left(\frac{m_B V_M}{M_B S}\right)^2\cdot\left(\frac{\Delta E_S}{\Delta E_\tau}\right)^2$$

in which τ denotes the constant current pulse time (s), m_B, V_M, M_B, and S represent the weight (g), molar volume (cm^3 mol^{-1}), molecular weight (g mol^{-1}) of the electrode and contacting area (cm^2) between the electrode and electrolyte, respectively.

S2 Supplementary Figures

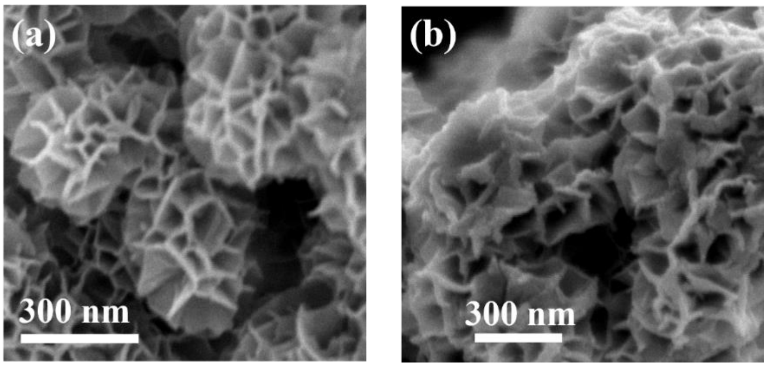

Fig. S1 a-b SEM images of δ-MnO_{2-x}-0.5 and δ-MnO_{2-x}-5.0

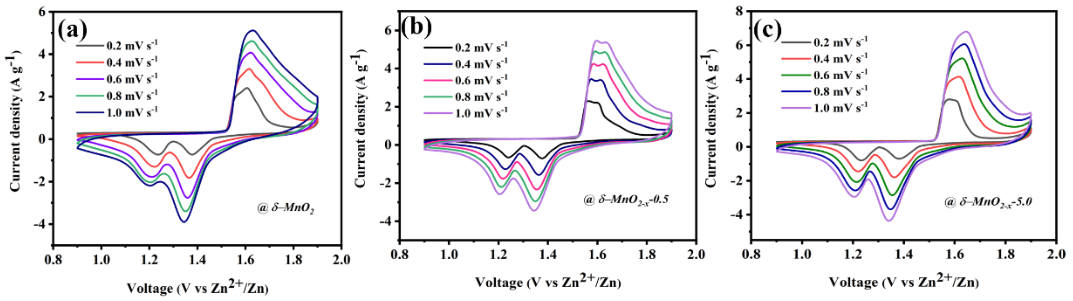

Fig. S2 CV curves for **a** δ-MnO_2, **b** δ-MnO_2-x-0.5 and **c** δ-$MnO_{2\text{-}x}$-5.0 electrodes at 0.2~2.0 mVs^{-1}

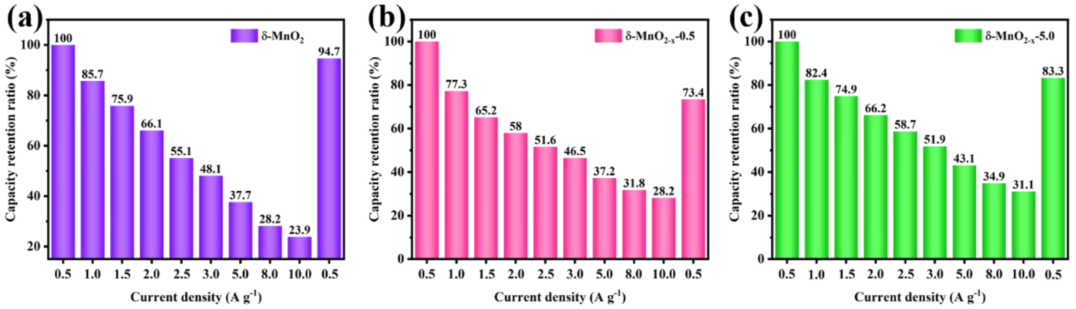

Fig. S3 Capacity retention rate of **a** δ-MnO_2, **b** δ-MnO_2-x-0.5 and **c** δ-$MnO_{2\text{-}x}$-5.0

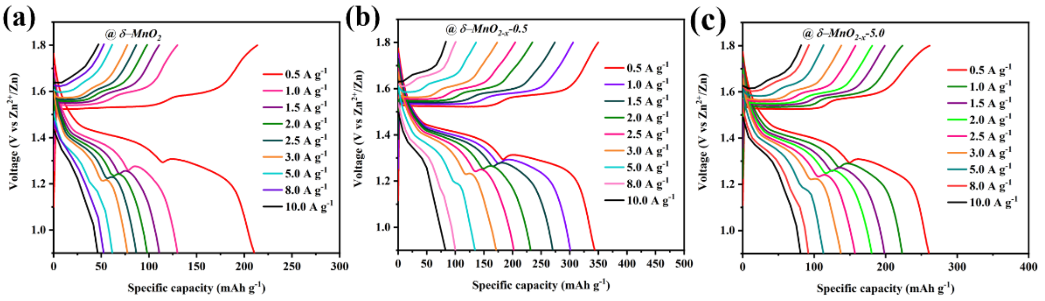

Fig. S4 Charge/discharge profiles of the **a** δ-MnO_2, **b** δ-$MnO_{2\text{-}x}$-0.5 and **c** δ-$MnO_{2\text{-}x}$-5.0 at 0.5~10.0 A g^{-1}

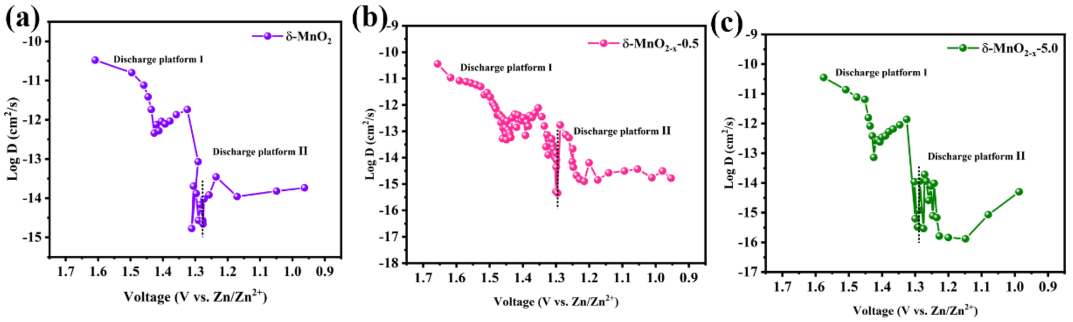

Fig. S5 In-situ ion diffusion coefficients of the **a** δ-MnO_2, **b** δ-$MnO_{2\text{-}x}$-0.5 and **c** δ-$MnO_{2\text{-}x}$-5.0

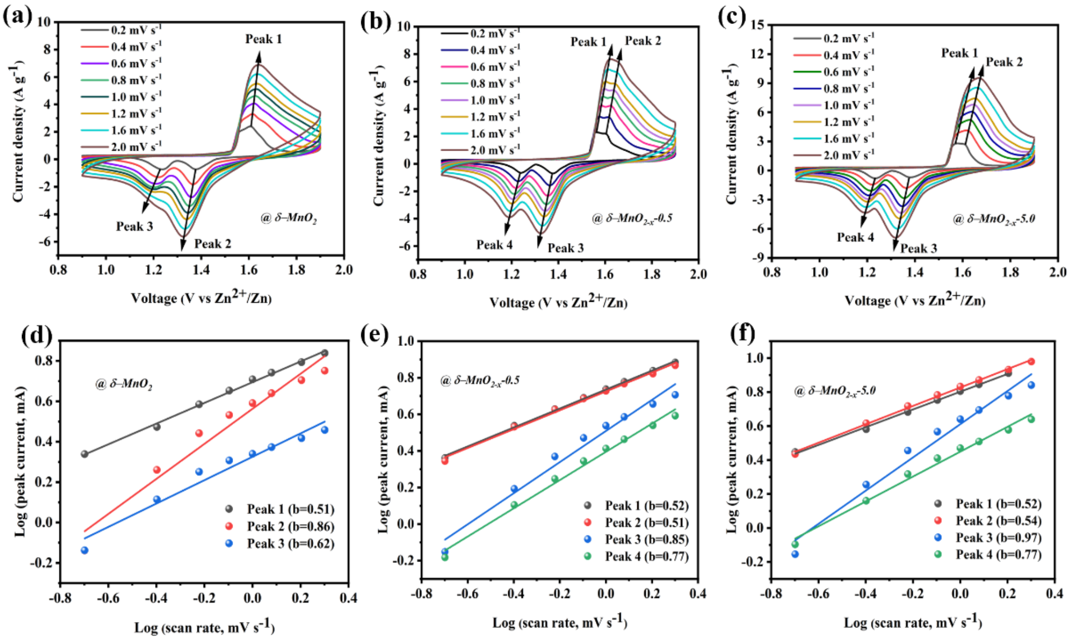

Fig. S6 CV profiles at diverse sweep rates of the **a** δ-MnO_2, **b** δ-MnO_{2-x}-0.5 and **c** δ-MnO_{2-x}-5.0. The evaluation of b values from linear fits in logarithmic plots of peak currents versus scan rates for the **d** δ-MnO_2, **e** δ-MnO_{2-x}-0.5 and **f** δ-MnO_{2-x}-5.0

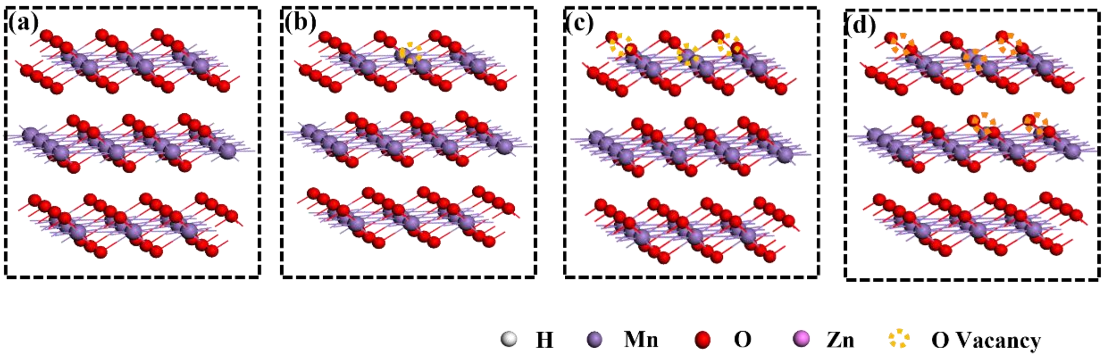

Fig. S7 The side-view theoretical models of **a** δ-MnO_2, **b** δ-MnO_{2-x}-0.5, **c** δ-MnO_{2-x}-2.0 and **d** δ-MnO_{2-x}-5.0

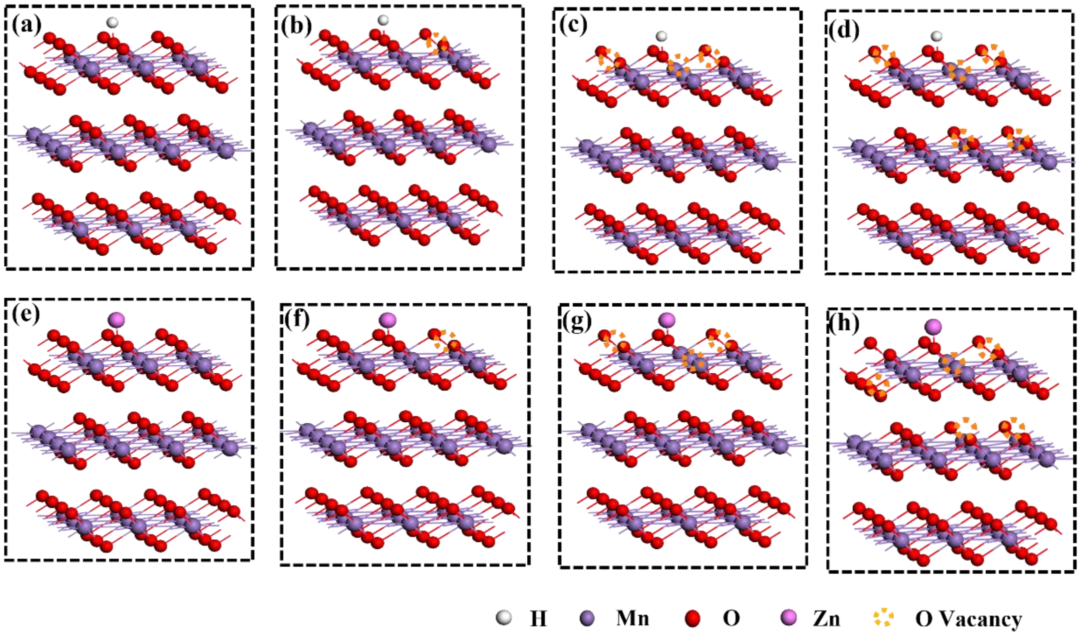

Fig. S8 a The side-view theoretical models of δ-MnO_2 adsorbing H^+, **b** δ-MnO_{2-x}-0.5 adsorbing H^+, **c** δ-MnO_{2-x}-2.0 adsorbing H^+, **d** δ-MnO_{2-x}-5.0 adsorbing H^+ and **e** δ-MnO_2 adsorbing Zn^{2+}, **f** δ-MnO_{2-x}-0.5 adsorbing Zn^{2+}, **g** δ-MnO_{2-x}-2.0 adsorbing Zn^{2+}, **h** δ-MnO_{2-x}-5.0 adsorbing Zn^{2+}

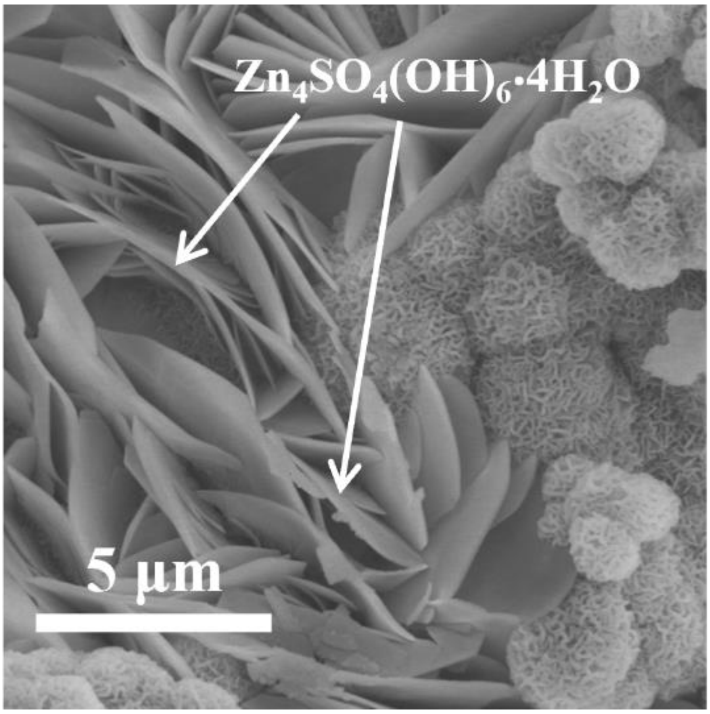

Fig. S9 The ex-situ FESEM image at fully discharged state (point V)

Table S1 The comparison of electrochemical properties of δ-MnO_{2-x}-2.0 with the previously reported cathode materials for zinc ion batteries

Cathode materials	Voltage range	Specific capacitance	Rate performance	References
$Mn_{0.15}V_2O_5·nH_2O$	0.2~1.6 V	299 mAh·g^{-1} @0.5 A·g^{-1}	150mAh·g^{-1} @10 A·g^{-1}	[S1]
$K_{0.43}(NH_4)_{0.12}V_2O_{5-δ}$	1.7~4.0 V	373.7 mAh·g^{-1} @0.5 A·g^{-1}	213 mAh·g^{-1} @10 A·g^{-1}	[S2]
P-Co-NVO	0.3~1.2 V	253.5 mAh·g^{-1} @0.5 A·g^{-1}	167.4 mAh·g^{-1} @10 A·g^{-1}	[S3]
V_2O_3	0.2~1.6 V	382.5 mAh·g^{-1} @0.4 A·g^{-1}	205 mAh·g^{-1} @12.8 A·g^{-1}	[S4]
O_d−HVO @PPy	0.2~1.6 V	346.5 mAh·g^{-1} @0.1 A·g^{-1}	252.6 mAh·g^{-1} @10 A·g^{-1}	[S5]
Na^+ doping VO_2 nanobelts (NVO)	0.2~1.6 V	345 mAh·g^{-1} @0.2 A·g^{-1}	121 mAh·g^{-1} @10 A·g^{-1}	[S6]
ZnO-QDs-VN-0.5	0.4~1.6 V	348.5 mAh·g^{-1} @0.2 A·g^{-1}	125.4 mAh·g-1 @5.0 A·g^{-1}	[S7]
MnO_2@PANI	1.0~1.85 V	342 mAh·g^{-1} @0.2 A·g^{-1}	100 mAh·g-1 @5.0 A·g^{-1}	[S8]
S-MnO_2	0.8~1.8 V	324 mAh·g^{-1} @0.2 A·g^{-1}	205 mAh·g^{-1} @2.0 A·g^{-1}	[S9]
δ-MnO_2 NDs	1.0~1.9 V	335 mAh·g^{-1} @0.1 A·g^{-1}	206 mAh·g^{-1} @2.0 A·g^{-1}	[S10]
**δ-MnO_{2-x}-2.0**	**0.9~1.8 V**	**551.8 mAh·g^{-1} @0.5 A·g^{-1}**	**420.7 mAh·g^{-1} @2.0 A·g^{-1} 328.6 mAh·g^{-1} @5.0 A·g^{-1} 262.2 mAh·g^{-1} @10.0 A·g^{-1}**	**This work**

Table S2 The comparison of electrochemical properties of δ-MnO_{2-x}-2.0 with the previously reported cathode materials for zinc ion batteries

Samples	Cycling current density	Cycle performance	References
Zn//β-MnO_2	0.2 A·g^{-1}	53.7% (after 1000 cycles)	[S11]
δ-MnO_2 NDs	1.0 A·g^{-1}	86.2% (after 1000 cycles)	[S10]
V_2O_5/CNTs hybrid paper	10.0 A·g^{-1}	76.9% (after 500 cycles)	[S12]
ZnO-QDs-VN-0.5	5.0 A·g^{-1}	54% (after 1800 cycles)	[S7]
ZNCMO@N-rGO	1.0 A·g^{-1}	78.5% (after 900 cycles)	[S13]
Zn/rGO // $V_3O_7·H_2O$/rGO	1.5 A·g^{-1}	79% (after 1000 cycles)	[S14]
NiHCF/RGO	0.2 A·g^{-1}	80.2% (after 1000 cycles)	[S15]
Zn/NVO	4.0 A·g^{-1}	82% (after 1000 cycles)	[S16]
β-MnO_2	1.0 A·g^{-1}	89.1% (after 600 cycles)	[S17]
MnO_2@PEDOT	1.11 A·g^{-1}	83.7% (after 300 cycles)	[S18]
**δ-MnO_{2-x}-2.0**	**3.0 A·g^{-1}**	**~90% (after 500cycles) ~87% (after 1000 cycles) ~83% (after 1500 cycles)**	**This work**

Supplementary References

[S1] H. Geng, M. Cheng, B. Wang, Y. Yang, Y. Zhang et al., Electronic structure regulation of layered vanadium oxide via interlayer doping strategy toward superior high-rate and low-temperature zinc-ion batteries. Adv. Funct. Mater. **30**, 112 (2019). https://doi.org/10.1002/adfm.201907684

[S2] Y. Zhao, S. Liang, X. Shi, Y. Yang, Y. Tang et al., Synergetic effect of alkali-site substitution and oxygen vacancy boosting vanadate cathode for super-stable potassium and zinc storage. Adv. Funct. Mater. **32**, 2203819 (2022). https://doi.org/10.1002/adfm.202203819

[S3] M. Du, Z. Miao, H. Li, F. Zhang, Y. Sang et al., Oxygen-vacancy and phosphate coordination triggered strain engineering of vanadium oxide for high-performance aqueous zinc ion storage. Nano Energy **89**, 106477(2021). https://doi.org/10.1016/j.nanoen.2021.106477

[S4] J. Ding, H. Zheng, H. Gao, Q. Liu, Z. Hu et al., In situ lattice tunnel distortion of vanadium trioxide for enhancing zinc ion storage. Adv. Energy Mater. **11**, 2100973 (2021). https://doi.org/10.1002/aenm.202100973

[S5] Z. Zhang, B. Xi, X. Wang, X. Ma, W. Chen et al., Oxygen defects engineering of $VO_2 \cdot xH_2O$ nanosheets via in situ polypyrrole polymerization for efficient aqueous zinc ion storage. Adv. Funct. Mater. **31**, 2103070 (2021). https://doi.org/10.1002/adfm.202103070

[S6] Y. Liu, X. Wu, Hydrogen and sodium ions Co-intercalated vanadium dioxide electrode materials with enhanced zinc ion storage capacity. Nano Energy **86**, 106124 (2021). https://doi.org/10.1016/j.nanoen.2021.106124

[S7] Y. Bai, H. Zhang, B. Xiang, Q. Yao, L. Dou et al., Engineering porous structure in Bi-component-active ZnO quantum dots anchored vanadium nitride boosts reaction kinetics for zinc storage. Nano Energy **89**, 106386 (2021). https://doi.org/10.1016/j.nanoen.2021.106386

[S8] N. Li, Z. Hou, S. Liang, Y. Cao, H. Liu et al., Highly flexible MnO_2@polyaniline core-shell nanowire film toward substantially expedited zinc energy storage. Chem. Eng. J. **452**, 139408 (2023). https://doi.org/10.1016/j.cej.2022.139408

[S9] Y. Zhao, P. Zhang, J. Liang, X. Xia, L. Ren et al., Uncovering sulfur doping effect in MnO_2 nanosheets as an efficient cathode for aqueous zinc ion battery. Energy Stor. Mater. **47**, 424-433 (2022). https://doi.org/10.1016/j.ensm.2022.02.030

[S10] H. Tang, W. Chen, N. Li, Z. Hu, L. Xiao et al., Layered MnO_2 nanodots as high-rate and stable cathode materials for aqueous zinc-ion storage. Energy Stor. Mater. **48**, 335-343 (2022). https://doi.org/10.1016/j.ensm.2022.03.042

[S11] W. Liu, X. Zhang, Y. Huang, B. Jiang, Z. Chang et al., β-MnO_2 with proton conversion mechanism in rechargeable zinc ion battery. J. Energy Chem. **56**, 365-373 (2021). https://doi.org/10.1016/j.jechem.2020.07.027

[S12] Y. Li, Z. Huang, P. K. Kalambate, Y. Zhong, Z. Huang et al., V_2O_5 nanopaper as a cathode material with high capacity and long cycle life for rechargeable aqueous zinc-ion battery. Nano Energy **60**, 752-759 (2019). https://doi.org/10.1016/j.nanoen.2019.04.009

[S13] Y. Tao, Z. Li, L. Tang, X. Pu, T. Cao et al., Nickel and cobalt co-substituted spinel $ZnMn_2O_4$@N-rGO for increased capacity and stability as a cathode material for rechargeable aqueous zinc-ion battery. Electrochim. Acta **331**,135296 (2020). https://doi.org/10.1016/j.electacta.2019.135296

[S14] C. Shen, X. Li, N. Li, K. Xie, J. G. Wang et al., Graphene-boosted, high-performance aqueous Zn-ion battery. ACS Appl. Mater. Interfaces **10**, 25446-25453 (2018). https://doi.org/10.1021/acsami.8b07781

[S15] Y. Xue, Y. Chen, X. Shen, A. Zhong, Z. Ji et al., Decoration of nickel hexacyanoferrate nanocubes onto reduced graphene oxide sheets as high-performance cathode material for rechargeable aqueous zinc-ion batteries. J. Colloid Interface Sci. **609**, 297-306 (2022). https://doi.org/10.1016/j.jcis.2021.12.014

[S16] F. Wan, L. Zhang, X. Dai, X. Wang, Z. Niu et al., Aqueous rechargeable zinc/sodium vanadate batteries with enhanced performance from simultaneous insertion of dual carriers. Nat. Commun. **9**, 1656 (2018). https://doi.org/10.1038/s41467-018-04060-8

[S17] X. Shi, G. Xu, S. Liang, C. Li, S. Guo et al., Homogeneous deposition of zinc on three-dimensional porous copper foam as a superior zinc metal anode. ACS Sustain. Chem. Eng. **7**, 17737-17746 (2019). https://doi.org/10.1021/acssuschemeng.9b04085

[S18] Y. Zeng, X. Zhang, Y. Meng, M. Yu, J. Yi et al., Achieving ultrahigh energy density and long durability in a flexible rechargeable quasi-solid-state Zn-MnO_2 battery. Adv. Mater. **29**, 1700274 (2017). https://doi.org/10.1002/adma.201700274
